# Effectiveness of an Emergency Department–Based Machine Learning Clinical Decision Support Tool to Prevent Outpatient Falls Among Older Adults: Protocol for a Quasi-Experimental Study

**DOI:** 10.2196/48128

**Published:** 2023-08-03

**Authors:** Daniel J Hekman, Amy L Cochran, Apoorva P Maru, Hanna J Barton, Manish N Shah, Douglas Wiegmann, Maureen A Smith, Frank Liao, Brian W Patterson

**Affiliations:** 1 BerbeeWalsh Department of Emergency Medicine University of Wisconsin-Madison Madison, WI United States; 2 Department of Population Health University of Wisconsin-Madison Madison, WI United States; 3 Department of Industrial and Systems Engineering University of Wisconsin-Madison Madison, WI United States; 4 Health Innovation Program University of Wisconsin-Madison Madison, WI United States; 5 Department of Applied Data Science UWHealth Hospitals and Clinics University of Wisconsin-Madison Madison, WI United States

**Keywords:** falls, emergency medicine, machine learning, clinical decision support, automated screening, geriatrics

## Abstract

**Background:**

Emergency department (ED) providers are important collaborators in preventing falls for older adults because they are often the first health care providers to see a patient after a fall and because at-home falls are often preceded by previous ED visits. Previous work has shown that ED referrals to falls interventions can reduce the risk of an at-home fall by 38%. Screening patients at risk for a fall can be time-consuming and difficult to implement in the ED setting. Machine learning (ML) and clinical decision support (CDS) offer the potential of automating the screening process. However, it remains unclear whether automation of screening and referrals can reduce the risk of future falls among older patients.

**Objective:**

The goal of this paper is to describe a research protocol for evaluating the effectiveness of an automated screening and referral intervention. These findings will inform ongoing discussions about the use of ML and artificial intelligence to augment medical decision-making.

**Methods:**

To assess the effectiveness of our program for patients receiving the falls risk intervention, our primary analysis will be to obtain referral completion rates at 3 different EDs. We will use a quasi-experimental design known as a sharp regression discontinuity with regard to intent-to-treat, since the intervention is administered to patients whose risk score falls above a threshold. A conditional logistic regression model will be built to describe 6-month fall risk at each site as a function of the intervention, patient demographics, and risk score. The odds ratio of a return visit for a fall and the 95% CI will be estimated by comparing those identified as high risk by the ML-based CDS (ML-CDS) and those who were not but had a similar risk profile.

**Results:**

The ML-CDS tool under study has been implemented at 2 of the 3 EDs in our study. As of April 2023, a total of 1326 patient encounters have been flagged for providers, and 339 unique patients have been referred to the mobility and falls clinic. To date, 15% (45/339) of patients have scheduled an appointment with the clinic.

**Conclusions:**

This study seeks to quantify the impact of an ML-CDS intervention on patient behavior and outcomes. Our end-to-end data set allows for a more meaningful analysis of patient outcomes than other studies focused on interim outcomes, and our multisite implementation plan will demonstrate applicability to a broad population and the possibility to adapt the intervention to other EDs and achieve similar results. Our statistical methodology, regression discontinuity design, allows for causal inference from observational data and a staggered implementation strategy allows for the identification of secular trends that could affect causal associations and allow mitigation as necessary.

**Trial Registration:**

ClinicalTrials.gov NCT05810064; https://www.clinicaltrials.gov/study/NCT05810064

**International Registered Report Identifier (IRRID):**

DERR1-10.2196/48128

## Introduction

Falls are highly prevalent among older adults in the United States [1,2] and are a common reason for emergency department (ED) visits [2-4]. They often precipitate a decline in the patient’s overall health and ability to perform activities of daily living, such as dressing, bathing, and toileting [3,5]. ED providers are important collaborators in preventing at-home falls because they are often the first providers to see a patient after a fall (secondary prevention) [1] and because outpatient falls are often preceded by previous ED visits (primary prevention) [6]. This phenomenon is especially true for patients being discharged from the ED [3].

Patients presenting to the ED are at higher risk of falls than the general population and are more likely to face barriers that limit their access to screening and interventions available in other health care settings [7-12]. While previous work has shown that ED referrals to falls prevention interventions can reduce the risk of an at-home fall by 38% [13], ED-based interventions to identify and refer high-risk older adults for falls prevention services have not been widely implemented [14]. Major barriers to fall risk screening in the ED include the additional time and resources necessary to perform in-person screening, but the integration of machine learning (ML) and clinical decision support (CDS) offers the possibility of automating the screening process, thereby making it feasible to incorporate in routine ED practice at scale without adding to provider cognitive burden [15-19]. However, it remains unclear whether the automation of such screening and referral systems can reduce the risk of future falls among older patients. The goal of this paper to is describe a research protocol for a study evaluating the effectiveness of an automated screening and referral intervention, both in terms of leading to evaluations at the Mobility and Falls Clinic and preventing future falls.

More generally, this study will provide data on the effectiveness of a model of an automated intervention to identify and refer patients during an ED visit. This model of care is extensible to many other conditions and situations in which ED patients would benefit from screening and primary or secondary prevention services.

## Methods

### Setting and Patient Population

This study takes place at 3 EDs, described in Table 1, within a health system in the Midwestern United States. All 3 EDs share an instance of the electronic health record (EHR) developed by Epic, though system configuration (eg, nursing flow sheets and order sets) is not identical at the 3 sites.

**Table 1:** Intervention settings.

ED^a^ site	Type	Intervention implementation date	Approximate annual volume of patients, n	Staffing
ED 1	Academic, level 1 trauma center	February 22, 2022	60,000 (25% geriatric)	Attending group 1, advanced practice providers, residents
ED 2	Community	September 27, 2022	22,000 (28% geriatric)	Attending group 1, advanced practice providers
ED 3	Community	Planned September 2023	65,000 (20% geriatric)	Attending group 2, advanced practice providers

^a^ED: emergency department.

The patient population for our study is individuals aged 65 years or older who present to participating EDs between February 22, 2022, and spring 2026, were discharged, and who had (at the time of ED visit) a primary care provider practicing within the health system in which this study is based. Because the ML-based CDS (ML-CDS) will be implemented in a rolling fashion at different EDs within the system, the start date for data will vary at different study sites. Dates were chosen to have at least two years’ worth of index ED data for each site and an additional 6 months of follow-up ED visits to identify subsequent falls.

### Intervention

These patients will be evaluated by our ML algorithm, and patients at high risk for an at-home fall within the next 6 months were flagged by our CDS when a provider documented a disposition of discharge from the ED [20,21]. The intervention, as well as training and validation of the algorithm, are limited to this in-system patient population for 2 reasons: first, these patients have a more complete patient history in the EHR for the algorithm to draw on, and second, this restriction mitigates the possibility that access to care or insurance coverage network would be a barrier to patients being seen in the falls clinic.

The study intervention applies automation to harness existing data resources and information systems both to estimate risk at the patient level and to facilitate referral among high-risk patients. This risk estimate drives a CDS tool that presents a referral recommendation to the physician within the ED workflow. This selective application of automation is aligned with human factors principles [21-24] and allows busy clinicians to improve patient care without interruption to existing workflows.

**Figure 1:**
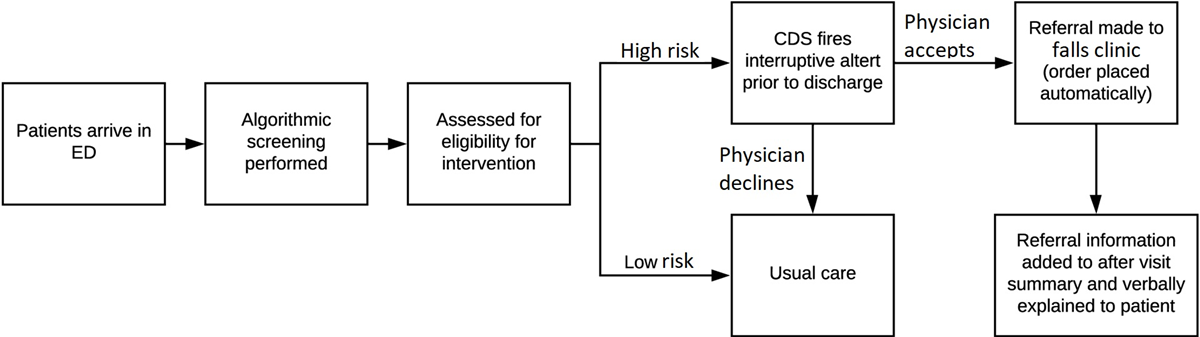
Operationalization of automated falls risk screening and referral process in the ED (adapted from Jacobsohn et al, reused with permission from Elsevier [21]). CDS: clinical decision support; ED: emergency department.

To support efforts by ED providers to prevent falls among older adults, our team has developed an ML-based CDS algorithm to identify the risk of a return to the ED for a fall for discharged patients [20]. This algorithm was subsequently incorporated into the EHR (Epic Systems). When providers are preparing to discharge a patient, this CDS alerts the ED provider if the ML algorithm identified the patient as at high risk of an at-home fall in the next 6 months and recommends referral to an outpatient mobility and falls clinic [21]. This process is illustrated in Figure 1 [21]. The ML algorithm will be retrained with the same feature set for each site prior to implementation. Implementation at each site will be done in compliance with institutional practice for CDS changes, following human factors engineering principles [23,24], and using best practices for ML-based CDS implementations [25].

At the outpatient clinic, patients receive an evidence-based, multidisciplinary intervention to help them reduce their risk of a fall [13]. Together, the algorithm and associated CDS tool form a novel automated screening and referral intervention that was designed and implemented using a human factors engineering framework to ensure both usability and an emphasis on patient safety [21-23].

### Outcomes

Our primary outcome is the rate of completed referrals to the Mobility and Falls Clinic among patients identified as high-risk. We define a completed referral as a patient completing an initial visit with a falls prevention specialist at the mobility and falls clinic.

Our secondary outcome is return visits to any ED for a fall within 6 months of the index ED visit, the outcome our ML algorithm was trained to predict. This outcome is meant to reflect the overall effectiveness of the intervention in preventing falls among patients identified as high-risk.

Additional process outcomes will be investigated to deconstruct steps from the identification of high-risk patients to completed referrals. These include whether a patient was referred to the outpatient mobility and falls clinic after being identified as high risk, whether referred patients were reached by schedulers, and whether referred patients scheduled an initial appointment, regardless of whether they attended.

### Data Collection

#### Variables

Variables for analysis were selected conceptually based on the Andersen behavioral model of health services use, a well-established model that provides a context for characterizing the many factors which lead to health care use [26-29]. Within this model, both contextual and individual factors influence patients’ initial ED visits and downstream health care use and outcomes. This model has been used to frame numerous prior studies involving ED use and falls among older adults [30,31], as well as the initial design and validation study of this intervention. During previous work, we have built a rich data set of covariates with over 700 potential EHR data features at ED 1 and will add data from ED 2 and ED 3 using the same data definitions [20]. These include patient factors, such as age, sex, race, ethnicity, and previous health system use; enabling factors, such as financial and physical availability of care; and ED encounter level factors, such as treatment team composition, ED length of stay, reason for visit, emergency severity index, diagnostic tests, and pharmaceutical and therapeutic interventions.

#### Data Retrieval Process

Data will be collected from the EHR in 4 steps. First, we will retrieve data about the patient, their index ED visit, and falls risk information—ML algorithm risk score, walking aids, history of falls, and acute care usage—from the EHR. Second, these data and information about referral orders and subsequent scheduled visits with the mobility and falls clinic will be uploaded to a Health Insurance Portability and Accountability Act (HIPAA)-compliant database built in a Research Electronic Data Capture (REDCap; Vanderbilt University) database [32]. This REDCap database is needed so documentation about the reason a patient did not ultimately schedule or attend the clinic visit can be abstracted. Specific reasons for not completing the referral are not always documented discretely, so they will be abstracted from free-text scheduler notes by 2 trained reviewers. Specifically, the reviewers will look for reasons the patient was documented to be ineligible. Reasons include whether the patient is in other physical therapy, in hospice, or in memory care; is too advanced to benefit, immobile, or wheelchair-bound; and whether the patient or caregiver declined or was unreachable. Third, after verifying outcome variables and the reason for not scheduling a visit, the data on the clinic visit will be joined with the curated data described above into a limited data set for analysis. Finally, falls outcome data will be merged with Medicare claims data to determine if the patient was treated for a fall at an outside ED.

### Statistical Analyses

#### Primary Outcome: Completed Referrals

The primary association of interest is whether an ED-based referral from an ML-based CDS can successfully cause patients to complete a specialist appointment. To assess the effectiveness of our program for patients receiving the falls risk intervention, our primary analysis will be to obtain point estimates and the 95% Wald CI of referral completion rate by site. We will perform a similar analysis to identify rates for prespecified process metrics, including referral orders placed among flagged patients and clinic appointments scheduled among at-risk patients. Further, we will perform a secondary analysis to identify subgroups at risk for lower odds of referral completion using a logistic regression model for each site to predict referral completion as a function of demography (sex, age, race, ethnicity, and education) and fall risk score. A model built for each site will account for site-level variability. Estimated coefficients and their 95% CIs will indicate risk factors for poor referral completion. This will help inform future implementation by, for example, identifying risk score thresholds for obtaining target rates of completed referrals for a given patient population.

#### Secondary Outcome: Return Visits to ED for a Fall

Although the intervention is not randomized, our study has a quasi-experimental design known as a sharp regression discontinuity with regards to intent-to-treat, since the intervention is administered to patients whose risk score falls above some threshold. This design allows for causal inference, provided that differences in outcomes for patients with similar risk scores can be attributed to the intervention (formally, expected potential outcomes are continuous in risk score). This occurs when patients are not manipulating their assignment to the intervention. Assuming this holds, we will primarily analyze only those patients whose risk scores fall within a band around the risk score threshold. Additionally, patients will be stratified by ED arrival date to account for changes to this threshold to be aligned with a sharp research continuity design. A conditional logistic regression model will be built to describe 6-month fall risk at each site as a function of the intervention, patient demographics, and risk score. The odds ratio of a return visit for fall and the 95% CI will be estimated by comparing those identified as high risk by the ML-CDS algorithm and those who were not but had a similar risk profile. Sensitivity analyses will be performed to examine the influence of bandwidth choice and key assumptions (ie, continuity of potential outcomes and linearity in risk score) on estimates. A secondary analysis will be to investigate the effect of covariates on completed referrals and falls using a stratified Cox proportional hazards model for time-to-fall events (right-censored by follow-up time).

#### Statistical Power

With the plan to flag 5 patients per week at each referral site, we plan to refer 520 patients per site, generating 1560 total patients referred. A sample size of 520 would lead to 95% Wald CI for referral completion rate by site that is 8.6% in width or smaller. This width provides sufficient precision for our study, considering that (1) a completion rate as small as 10% would be clinically meaningful and (2) we expect to observe a referral completion rate of 50% or higher—in which case, there would be nearly 100% power to reject a referral completion rate of 10% at a significance level of .05 when n=520.

For reference, very few patients were referred from the ED to the mobility and falls clinic prior to our intervention, and the clinic estimates that 80% of their patients referred from other providers visit the clinic. Power can be estimated for detecting a significant intervention effect on fall risk, provided additional covariates are ignored. In this case, a score test for this effect is equivalent to a Cochran-Mantel-Haenszel test of independence in a stratified sample. Such a test has 89.7% power to detect a difference in fall risk of 10% in a stratified sample size of 12 groups (in twelve 8-week periods over 2 years) assuming (1) a significance level of .05, (2) 40 patients receive the intervention and 40 patients do not who have similar risk scores, and (3) an average fall risk of 25%. For reference, our preliminary work predicted a number to treat of about 10 (ie, a fall risk difference of 10%) when setting the risk score threshold so that 5 persons per week receive the intervention (or 40 persons per 8 weeks) and an average fall risk of about 25% for individuals with a fall risk score above or near the fall risk score threshold. We plan on exploratory analysis of pooled data to evaluate the effect of specific covariates on rates of referral; in these cases, power will be lower based on the incidence of specific covariates in the data.

### Data Protection

The major potential risk to subjects is that of loss of confidentiality. We have policies and procedures in place to protect the confidentiality and security of patient data, and our data protection measures (for protected health information) are consistent with HIPAA privacy and security rules. Only persons directly involved in the project will have access to patient data. Access to computer-stored information will be strictly controlled with data stored on servers physically sequestered and protected behind both physical and digital access controls. All servers are behind our organization’s HIPAA firewall. All project personnel have successfully completed institutionally required human subjects training (required every 3 years) and HIPAA privacy and security training (required every year).

### Dissemination

Our multidisciplinary team spans emergency medicine, engineering, health services research, and biostatistics, facilitating dissemination to a diverse and multidisciplinary audience. We will leverage institutional resources across the health system and university and capitalize on the breadth of professional networks offered by our interdisciplinary team to ensure broad dissemination. Dissemination of findings and of the final intervention will be accomplished through 2 channels. Academic dissemination will occur through publication in relevant peer-reviewed journals and presentations at national conferences focused on aging research, informatics, human factors, and emergency medicine. We will also share findings at end-user and EHR-focused conferences to reach potential adopters who might not be reached by more formal outlets.

### Ethics Approval

This research plan was reviewed and approved by the University of Wisconsin Health Sciences Institutional Review Board (2021-0776). This observational quasi-experiment is registered with clinicaltrials.gov (NCT05810064).

## Results

As of April 30, 2023, the CDS tool has been implemented at 2 of the 3 sites. Providers at ED 1 have written 303 referral orders for 260 unique patients, or 5 referrals per week on average, which is our goal. ED 2, the community ED, has not been live with the ML-CDS as long, and providers have written 91 referral orders for 86 different patients, or just over 3 referrals per week. Both EDs refer roughly 30% of patient encounters the algorithm identifies: 303 referrals for 1007 patients flagged at ED 1 and 91 referrals for 319 patients flagged at ED 2. The patient populations between these 2 EDs have substantial overlap, and 6 patients have received referrals from both EDs. After conversations with the mobility and falls clinic, we do not see repeat referrals as a problem unless the patient has already completed 1 of the referrals, so our ML-CDS tool was updated to exclude patients with a completed or scheduled appointment at the mobility and falls clinic. Notably, the patient uptake of the final intervention is about 15% of patients referred—45 patients of 339 unique patients; 1 patient was seen at and referred by both ED 1 and 2—which is less than the 80% that the clinic estimates for other referral sources. While not surprising that uptake would be lower from ED provider referrals than from primary care provider referrals, this conversion rate is lower than we anticipated, and we have taken steps to improve messaging, patient follow-up, and providers’ ability to talk to patients about the program, and we have taken other steps that have shown some improvement appointment conversion in recent months. Additionally, waning concerns about COVID-19 have seemed to increase patients’ willingness to consider completing the referral.

## Discussion

### Hypothesis and Expected Findings

We expect that this research will show the beneficial impact of automated screenings on both patients receiving recommended care (completed referrals) and preventing adverse outcomes (falls). Even for patients who are not referred, we anticipate providers having conversations with patients who are flagged as high risk for a fall, which could still lead to increased compliance with ongoing treatment or seeking falls prevention treatment outside our partner clinic (eg, physical therapy available at an assisted living facility). These findings will inform ongoing discussions about the use of ML and artificial intelligence to augment medical decision-making.

### Significance of Principal Findings

Previous work with ML-CDS tools has focused on their implementation in a specific context [33] or on the predictive performance [34] of the underlying algorithm. Our unique data set will include information about patient’s health history (eg, health care use) prior to the ED index visit, referral completion, and subsequent ED return visits for falls (either in the system or from Medicare claims), providing a more complete picture of a given patient’s trajectory than most studies. We will use this unique data set to track not just ML-CDS performance in the context of its implementation, but in the context of larger patient health outcomes, which, to our knowledge, has not been done for this kind of long-term, preventative ML-CDS system. Similarly, the multisite implementation plan will demonstrate applicability to a broad population and the possibility to adapt the intervention to other EDs and achieve similar results.

Our statistical methodology (a regression discontinuity design) allows for an intent-to-treat causal inference about the impact flagging high-risk patients can have on patients’ future falls. Conversations with patients about the referral, even if a referral is not written, could have impacts on patients’ risk awareness and compliance with physical therapy, so ML-CDS flagging is the correct epidemiologic exposure to assess with respect to the outcomes of completed mobility and falls visits or return ED visits for falls. A staggered implementation strategy allows for the identification of secular trends that could affect causal association and mitigation as necessary.

### Limitations

To achieve the desired number of clinic referrals per week, the falls risk threshold for the CDS identification will be changed over time. Because regression discontinuity designs typically assume a static threshold for analysis, we will need to approach the regression discontinuity analysis carefully and with sufficient sensitivity analyses to ensure that these threshold changes do not compromise the findings. Simulation studies will be conducted to ensure the assumptions of regression discontinuity analysis are met.

Clinically, our assumptions about the effectiveness of the mobility and falls clinic intervention are based on PROFET (Prevention of Falls in the Elderly Trial) [13], which specifically evaluated patients referred from the ED. The intervention is based on evidence from a randomized clinical trial, but the magnitude of the effect may be different in our patient population—for example, due to endogenous factors like demographic composition of the community or exogenous factors related to changes in care patterns during the COVID-19 pandemic. In addition to methodological concerns, there are some pragmatic limitations. First, the intervention was implemented during the COVID-19 pandemic. During the study period and community, there were—and could continue to be—multiple waves of elevated SARS-CoV-2 infections and case counts that may dissuade some patients from scheduling an appointment at the mobility and falls clinic. Similarly, in order to maximize patient impact and to study the human factors inputs to this system, efforts were made to increase referral rates and referral completion with providers and patients. While these efforts will likely increase the statistical power over time, it is also likely that there will be secular effects in the imperfect compliance with referral rates and referral completion rates that are present in the data as a result of these efforts.

## Appendix

Multimedia Appendix 1 Summary Statement and Peer Review from Healthcare Information Technology Research (HITR) - Agency for Healthcare Research and Quality (USA).

## Abbreviations

CDS: clinical decision support
ED: emergency department
EHR: electronic health record
HIPAA: Health Insurance Portability and Accountability Act
ML: machine learning
ML-CDS: machine learning–based clinical decision support
PROFET: Prevention of Falls in the Elderly Trial
