# Supplementary material for: Effectiveness of an Emergency Department–Based Machine Learning Clinical Decision Support Tool to Prevent Outpatient Falls Among Older Adults: Protocol for a Quasi-Experimental Study
Source: JMIR Res Protoc. 2023 Aug 3;12:e48128. doi: 10.2196/48128 (PMC10436111; doi:10.2196/48128)
Supplement: Multimedia Appendix 1 [file resprot_v12i1e48128_app1.pdf]

SUMMARY STATEMENT
(Privileged Communication)

PROGRAM CONTACT:
Brent Sandmeyer
301-427-1441
brent.sandmeyer@ahrq.hhs.go
v

Release Date: 03/05/2021
Revised Date:

Application Number: 1 R18 HS027735-01A1
Formerly: 1R18HS027735-01

Principal Investigator

PATTERSON, BRIAN W

Applicant Organization: UNIVERSITY OF WISCONSIN-MADISON

Review Group: HITR
Healthcare Information Technology Research

Meeting Date: 02/25/2021
Council: MAY 2021
Requested Start: 07/01/2021

RFA/PA: PA18-793
PCC: CEPI

Project Title: Preventing Future Falls in Older Adult ED Patients: Evaluating the Implementation and Effectiveness of a Novel Automated Screening and Referral Intervention
SRG Action: Impact Score:20 Percentile:2 +

Human Subjects: 30-Human subjects involved - Certified, no SRG concerns
Animal Subjects: 10-No live vertebrate animals involved for competing appl.
Gender: 1A-Both genders, scientifically acceptable
Minority: 1A-Minorities and non-minorities, scientifically acceptable
Clinical Research - not NIH-defined Phase III Trial

Project Year	Direct Costs Requested	Estimated Total Cost
1	257,076	399,753
2	257,040	399,697
3	257,190	399,930
4	257,228	399,990
5	257,187	399,926
TOTAL	1,285,721	1,999,296

ADMINISTRATIVE BUDGET NOTE: The budget shown is the requested budget and has not been adjusted to reflect any recommendations made by reviewers. If an award is planned, the costs will be calculated by Institute grants management staff based on the recommendations outlined below in the COMMITTEE BUDGET RECOMMENDATIONS section.

RESUME AND SUMMARY OF DISCUSSION: This R18 health services research grant resubmission from Dr. Brian Patterson, from the University of Wisconsin-Madison, proposes to adapt the design of an automated screening and referral intervention for implementation in three diverse emergency departments (EDs) settings, and test the effectiveness of the automated screening and referral intervention on both completed referrals to a multidisciplinary fall prevention clinic and rates of injurious falls. The study will also evaluate implementation of the automated screening and referral intervention in three diverse ED sites using a mixed methods approach. The reviewers agreed on the significance of this study and noted that falls are an extremely significant problem in the community and the ED is a good place to identify patients in danger and refer. Identifying patients at high risk for falls when they present in the ED could prevent falls thus improving patient outcomes and reducing costs. They also noted that this is a well-written resubmission that will build upon pilot work to address the implementation of a clinical decision support tool using human factors and implementation science methods. The use of multiple datasets (collected data, Medicare claims, etc.) minimizes the risk of lost patients and may improve the algorithm. It includes a complimentary team of researchers with prior history of successfully working together. The resubmission is responsive to the previous review critique including issues with unspecified approach data collection and analysis methods. However, the reviewers also noted some very small concerns. There are some missing details about the EHR data analyses and implementation outside the investigators health system. The reviewers also noted an unclear description of the intersection between the clinical decision support (CDS) and the provider and why integration into workflow was previously a problem. Overall, the reviewers recommended this application for further consideration with an outstanding level of enthusiasm.

DESCRIPTION (provided by applicant): Falls are the leading traumatic cause of both injury and death among older adults. American emergency departments (EDs) see over 3 million fall victims yearly, yet they play little role in primary or secondary fall prevention. The ED is an ideal site to identify patients at risk of future falls, however in this setting preventive care cannot be implemented at the expense of the primary mission of the ED: the provision of emergency care in a time-pressured environment. As the population ages, and the ED continues to expand its role as the primary site for delivery of acute unscheduled care, there is an urgent need to create a scalable intervention to assess older adults for fall risk and link them to appropriate risk reduction interventions after discharge without adding additional workload for nurses or physicians. Through an AHRQ K08, our study team has developed and validated an innovative automated screening and referral intervention for fall risk. This intervention harnesses existing data to select and connect patients to appropriate primary and secondary prevention services after ED visits without adding burden to nurse or physician workloads. This intervention features smart use of automation for screening and referral tasks maintaining physician decision autonomy, as well as the unique ability to adjust referral rates based on clinic availability. This intervention features smart use of automation for screening and referral tasks maintaining physician decision autonomy, as well as the unique ability to adjust referral rates based on clinic availability. Based on our work, UW Health is currently piloting the intervention, and has committed to implementing it at three diverse ED sites. This study will adapt the intervention for implementation at additional sites, and investigates the implementation and effectiveness of the automated screening and referral process in all three EDs through three specific aims: 1) Adapt the design of an automated screening and referral intervention for implementation in three diverse ED settings, using a human factors approach. 2) Test the effectiveness of the automated screening and referral intervention on both completed referrals to a multidisciplinary fall prevention clinic and rates of injurious falls using EHR data generated during implementation. 3) Evaluate implementation of the automated screening and referral intervention in three diverse ED sites using a mixed methods approach. This grant proposal builds upon our previous innovative work developing both CDS and risk- stratification algorithms to improve the quality and safety of care delivered to older adult ED patients. We will address the urgent and growing need for a scalable strategy for fall risk reduction from the ED by demonstrating the effectiveness of our novel

approach in a study spanning diverse hospital types and patient populations. Furthermore, knowledge gained from this work will inform other use cases which could benefit from automated risk-stratification and care coordination in the ED and beyond.

PUBLIC HEALTH RELEVANCE: This proposal directly addresses the public health burden of the rising rates of significant falls in older adults which are the leading cause of traumatic morbidity and mortality in the elderly. Specifically, the use of an automated screening tool harnesses existing data resources and information systems to use the ED to better coordinate care between the ED and existing preventive resources in the health system. Beyond improving care for older adults with falls, this study will demonstrate the promise of using information technology to deliver public health interventions in the ED setting without diverting provider resources from the core ED mission of providing quality acute care.

CRITIQUE NOTE: The sections that follow are the essentially unedited, verbatim comments of the individual committee members assigned to review this application. The attached commentaries may not necessarily reflect the position of the reviewers at the close of group discussion, nor the final majority opinion of the group. The above RESUME/SUMMARY OF DISCUSSION represents the evaluation of the application by the entire committee.

CRITIQUE 1

Significance:	2
Investigator(s):	1
Innovation:	1
Approach:	3
Environment:	1

Overall Impact:
Strengths
- Well-written grant that will build upon pilot work to address the implementation of a clinical decision support tool using human factors and implementation science methods.

Weaknesses
- Some missing details about the EHR data analyses and implementation outside the investigators health system.

1. Significance:
Strengths
- Falls are a common source of morbidity in older adults and efforts to reduce falls through primary care has had limited success.

Weaknesses
- Investigators gloss over the future challenges associated with the dissemination of their approach outside their health system, which if this cannot be addressed would decrease the significance of their system.

2. Investigators:
Strengths
- Team seems to have the right combination of expertise necessary to complete the project.
- The PI is a K08 recipient and this work is a clear extension of his previous research.
- Team members have collaborated in the past.

Weaknesses
- Concern that Dr. Liao may have too little effort to assist with the implementation of the intervention at 2 additional sites within their health system.

3. Innovation:
Strengths
- Interventions aimed at targeting patients in the ED for prevention of future health outcomes is relatively novel, especially given that the intervention will need very little effort from limited time of ED personnel.
- Providing absolute risk predictions with the ability to titrate the level at which the intervention is triggered is not generally done and provides flexibility.

Weaknesses
- None noted.

4. Approach:
Strengths
- Nice use of existing theoretical models (i.e. RE-AIM and SEIPS) to drive the methodological techniques for evaluating the design and implementation of the tool.
- Robust use of Human Factors Design methodology to obtain feedback from clinicians, interdisciplinary team members, automated referrals, UW patient and Family Advisory council, etc.
- Investigators appear to have adequately addressed a previous reviewers concern about the distance between one of the sites and the Falls clinic.
- CMS claims data will be used to address previous concerns about injurious falls occurring outside the health system.

Weaknesses
- Some missing details about EHR data handling and future implementation.
- How were missing EHR data points handled in the regression modeling and how will missing EHR data points be handled when implemented in future patients?
- Lack of details about how the risk model calculation, which appears to occur outside of the EHR is "piped back into the EHR". Does it use FHIR resources or other APIs, exactly where do the results appear in the EHR and in what format? Is there an ETL process in place for storing the results in the EHR back up database?
- What happens when the cloud based system "goes down"?
- No apparent method for addressing the competing risk of death due to other causes during the follow up period. The project does not appear to take use regional death registries or tools like the National Death Index.
- Measures of SDoH are limited. For example, EHR addresses could be geocoded and mapped at the block group level with ACS data.

5. Environment:
Strengths
- Excellent academic university with high volume EDs that serve heterogeneous populations.
- Existing resources such as the ICTR, falls clinic, and advanced data science group.
- Established EHR system with data mapped to i2b2.
- Current use of a cloud computing system that can provide real-time calculations at the point of care.

Weaknesses
- None noted.

Protections for Human Subjects: Acceptable.
Strengths
- Appropriate data safeguards.
- Appropriate consent process.

Weaknesses
- None noted.

Single IRB for Cooperative Research
- NA

Degree of Responsiveness: Addresses public health burden of falls in older adults. Improves coordination of care. Demonstrates the use of IT to deliver better quality care.

Inclusion of Women, Minorities, and Individuals Across the Lifespan: Acceptable.
Strengths
- Includes women and minorities.
- Appropriately focused on older adults at risk for falling.

Weaknesses
- None noted.

Inclusion of AHRQ Priority Populations: Acceptable.
Strengths
- Includes an ED that serves a rural population and lower income individuals in both urban and rural settings.

Weaknesses
- None noted.

Budget and Period of Support: Appropriate, no concerns.

Data Management Plan: Adequate.

Resubmission Applications:
Strengths
- Addressed issues regarding distance of ED with falls clinic.
- Addressed issue regarding falls occurring outside the health system.

Weaknesses
- Did not address comments about missing EHR data and EHR connections.

CRITIQUE 2

Significance:	1
Investigator(s):	1
Innovation:	2
Approach:	2
Environment:	1

Overall Impact:
Strengths

- Identifying patients at high risk for falls when they present in the ED could prevent falls thus improving patient outcomes and reducing costs.
- Use of multiple datasets (collected data, Medicare claims, etc.) minimizes the risk of lost patients and may improve the algorithm.

Weaknesses

- COVID-19 may impact the number of patients who come to the ED and thus patients receiving/completing referrals.
- It is not clear how readily the algorithm can translate to other facilities with a different EHR.

1. Significance:

Strengths

- Falls are a significant issue for older patients and the healthcare system, leading to morbidity/mortality and high costs for care.
- Using the ED for prevention could shift care dynamics and prevent more costly care in the long term.

Weaknesses

- None noted.

2. Investigators:

Strengths

- Team is diverse in expertise and has strong experience.
- Statistician on the team.
- Established collaboration between team members.

Weaknesses

- None noted.

3. Innovation:

Strengths

- The adjustment threshold for referrals is interesting and novel.
- Use of the ED for prevention is unique.
- The predictive algorithm is fairly novel.

Weaknesses

- Methods are well established.

4. Approach:

Strengths

- Site selection supports capturing data from diverse populations.
- Linking Medicare claims data will help minimize lost data.
- Prior work suggests feasibility and appropriateness of the proposed methods.
- Potential problems and alternative strategies are described.

Weaknesses

- There is a potential that ED visits decreased due to COVID-19 as people seek telehealth and other options. It is not clear if that will have an impact on the proposed work.

5. Environment:

Strengths

- The environment is well positioned for the proposed work to be successful.
- The Mobility and Falls Clinic is a unique site for the intervention.
- EDs see a large number of patients.

Weaknesses

- None noted.

Protections for Human Subjects: Acceptable.
Strengths
- Protections are appropriate for potential participants.

Weaknesses
- None noted.

Single IRB for Cooperative Research: N/A.

Degree of Responsiveness: Highly Responsive.
Strengths
- The application seeks to improve patient safety related to potential falls through predictive use of data.

Weaknesses
- None noted.

Inclusion of Women, Minorities, and Individuals Across the Lifespan: Acceptable**.**
Strengths
- Participants will be recruited to ensure inclusion of women and minorities.

Weaknesses
- None noted.

Inclusion of AHRQ Priority Populations: Acceptable.
Strengths
- Focus is on the elderly population.

Weaknesses
- None noted.

Budget and Period of Support: Acceptable.
Strengths
- Budget and period of support are reasonable for the proposed work.

Weaknesses
- None noted.

Data Management Plan: Adequate.

Resubmission Applications:
Strengths
- Concerns about missing data/patients lost to follow-up have been addressed.
- Greater detail is provided about qualitative analysis.
- Letter of support now indicates capacity for number of potential referrals.

Weaknesses
- None noted.

CRITIQUE 3

Significance:	1
Investigator(s):	2

Innovation:	1
Approach:	2
Environment:	1

Overall Impact:
Strengths
- Falls are an extremely significant problem in the community and the ED is a good place to identify patients in danger and refer.
- Complimentary team of researchers with prior history of successfully working together.
- PI has successfully completed related research and has extensive knowledge of the available information system sources.
- Application is responsive to some the previous review critique related to unspecified approach data collection and analysis methods.

Weaknesses
- Vague description of the intersection between the CDS and the provider and why integration into workflow was previously a problem.
- Not clear how the provider will be alerted and respond to a CDS alert for fall risk and referral.

1. Significance:
Strengths
- Addresses a significant problem, falls in the elderly, where not screening for in the ED is a missed opportunity.

Weaknesses
- None noted.

2. Investigators:
Strengths
- Complimentary team of researchers with prior history of successfully working together.
- PI has successfully completed related research and has extensive knowledge of the available information system sources.

Weaknesses
- None noted.

3. Innovation:
Strengths
- Innovative to screen and refer in the ED using existing data.

Weaknesses
- Better method for screening and referral follow-up could be described.

4. Approach:
Strengths
- Responsive to previous critique regarding need for better specification of data collection and analysis.
- Detailed explanation of qualitative coding strategy.
- Detailed timeline of activities provided.
- Adequately address human subjects recruitment and protections.

Weaknesses
- Not clear how the CDS tool will be implemented in the workflow. Even though this is to be developed based on participant feedback, some idea of CDS/provider interface would be helpful.

5. Environment:
Strengths
- Strong environment with adequate support and resources.

Weaknesses
- None noted.

Protections for Human Subjects: Acceptable.

Degree of Responsiveness: Responsive to FOA and AHRQ priorities.

Inclusion of Women, Minorities, and Individuals Across the Lifespan: Acceptable.

Inclusion of AHRQ Priority Populations: Acceptable.

Budget and Period of Support: Acceptable.

Data Management Plan: Adequate.

Resubmission Applications: Mostly responsive to previous critiques.

THE FOLLOWING RESUME SECTIONS WERE PREPARED BY THE SCIENTIFIC REVIEW ADMINISTRATOR TO SUMMARIZE THE OUTCOME OF DISCUSSIONS OF THE REVIEW COMMITTEE ON THE FOLLOWING ISSUES:

PROTECTION OF HUMAN SUBJECTS (Resume): ACCEPTABLE.

INCLUSION OF WOMEN PLAN (Resume): ACCEPTABLE.

INCLUSION OF MINORITIES PLAN (Resume): ACCEPTABLE.

INCLUSION OF AHRQ PRIORITY POPULATIONS PLAN (Resume): ACCEPTABLE.

COMMITTEE BUDGET RECOMMENDATIONS: The budget was recommended as requested.

Footnotes for 1 R18 HS027735-01A1; PI Name: Patterson, Brian W

+ Derived from the range of percentile values calculated for the study section that reviewed this application.

MEETING ROSTER

Healthcare Information Technology Research
AGENCY FOR HEALTHCARE RESEARCH AND QUALITY
HITR
02/25/2021 - 02/26/2021

CHAIRPERSON(S)

JENDERS, ROBERT ALLEN, BS, MD, MS
PROFESSOR OF MEDICINE AND CO-DIRECTOR
CENTER FOR BIOMEDICAL INFORMATICS
CHARLES DREW UNIVERSITY, SCHOOL OF MEDICINE
SCHOOL OF MEDICINE
LOS ANGELES, CA 90059

MEMBERS

ASAN, ONUR, PHD
ASSOCIATE PROFESSOR
SCHOOL OF SYSTEMS AND ENTERPRISES
STEVENS INSTITUTE OF TECHNOLOGY, NEW JERSEY
HOBOKEN, NJ 07030

ASCHE, CARL, PHD, MSC, MS
PROFESSOR
DEPARTMENT OF MEDICINE
UNIVERSITY OF ILLINOIS
ONE ILLINI DRIVE
PEORIA, IL 61605

BIAGIOLI, FRANCES EMILY, MD
PROFESSOR AND VICE CHAIR FOR EDUCATION
OREGON HEALTH & SCIENCE UNIVERSITY (OHSU)
DEPARTMENT OF FAMILY MEDIICINE
SCHOOL OF MEDCINE
PORTLAND, OR 97239

CANTOR, MICHAEL, MD, MA
EXECUTIVE DIRECTOR
CLINICAL INFORMATICS
REGENERON GENETICS CENTER
TARRYTOWN, NY 10591

CARON, ALEECE, PHD
ASSOCIATE PROFESSOR OF MEDICINE
CENTER FOR HEALTHCARE, RESEARCH, AND POLICY
THE METROHEALTH MEDICAL CENTER
CASE WESTERN RESERVE UNIVERSITY
CLEVELAND, OH 44109

CHUI, MICHELLE ANNE, PHD, PHMD
HAMMEL SANDERS PROFESSOR
SOCIAL & ADMINISTRATIVE SCIENCES
SCHOOL OF PHARMACY
UNIVERSITY OF WISCONSIN
MADISON, WI 53705

FRIEDMAN, CHARLES P, PHD
JOSIAH MACEY JR. PROFESSOR AND CHAIR
HEALTH INFORMATICS PROGRAM
DEPARTMENT OF LEARNING AND HEALTH SCIENCES
UNIVERSITY OF MICHIGAN MEDICAL SCHOOL
ANN ARBOR, MI 48109

KOZALINSKI, REBECCA SUSAN, PHD, MS, BSN
ASSOCIATE PROFESSOR
OUHSC CON
UNIVERSITY OF OKLAHOMA
OKLAHOMA CITY, OK 73117

LANHAM, HOLLY J., PHD, MBA
ASSOCIATE PROFESSOR
DEPARTMENT OF MEDICINE
DIVISION OF GENERAL AND HOSPITAL MEDICINE
UNIVERSITY OF TEXAS HEALTH SAN ANTONIO
SAN ANTONIO, TX 78229

LEROY, GONDY, PHD
ELLER FELLOW AND ASSOCIATE PROFESSOR OF MIS,
PRESIDENT OF AIS SIG-HEALTH
MANAGEMENT INFORMATION SYSTEMS
ELLER COLLEGE OF MANAGEMENT
UNIVERSITY OF ARIZONA
TUCSON, AZ 85721

MELTON, BRITTANY LEE, PHD, PHMD
ASSOCIATE PROFESSOR
DEPARTMENT OF PHARMACY PRACTICE
UNIVERSITY OF KANSAS
KANSAS CITY, KS 66160

MELTON, KRISTIN R, MD
ASSOCIATE PROFESSOR OF PEDIATRICS
DIVISION OF NEONATOLOGY, PULMONARY AND
PERINATAL BIOLOGY
UNIVERSITY OF CINCINNATI
CINCINNATI, OH 45229

MOSS, JACQUELINE A, PHD, FAAN, RN
PROFESSOR AND ASSOCIATE DEAN FOR TECHNOLOGY &
INNOVATION
SCHOOL OF NURSING
UNIVERSITY OF ALABAMA AT BIRMINGHAM
HOOVER, AL 35244

MOYE-DICKERSON, PAMELA, PHMD
CLINICAL ASSOCIATE PROFESSOR
PHARMACY PRACTICE/COLLEGE OF PHARMACY
MERCER UNIVERSITY
ATLANTA, GA 30341

RAO, GOUTHAM, MD
PROFESSOR AND CHAIRMAN
FAMILY MEDICINE & COMMUNITY HEALTH
CASE WESTERN RESERVE UNIVERSITY &
UNIVERSITY HOSPITALS OF CLEVELAND
CLEVELAND, OH 44106

REED, MARY, DRPH
RESEARCH SCIENTIST
DIVISION OF RESEARCH
KAISER PERMANENTE
NORTHERN CALIFONIA
OAKLAND, CA 94610

ROSSETTI, SARAH COLLINS, PHD, RN
ASSISTANT PROFESSOR OF BIOMEDICAL INFORMATICS
AND NURSING
DEPARTMENT OF BIOMEDICAL INFORMATICS
SCHOOL OF NURSING
COLUMBIA UNIVERSITY MEDICAL CENTER
NEW YORK, NY 10032

SORONDO, BARBARA, MBA, MD
DIRECTOR
CLINICAL DEVELOPMENT
LG CHEM LIFE SCIENCES
INNOVATION CENTER, INC.
CAMBRIDGE, MA 02142

TABER, DAVID J., PHMD, MS
PROFESSOR
DEPARTMENT OF SURGERY
DIVISION OF TRANSPLANT SURGERY
MEDICAL UNIVERSITY OF SOUTH CAROLINA
CHARLESTON, SC 29425

WELLS, BRIAN JAY, MD, PHD
ASSOCIATE PROFESSOR
DEPARTMENT OF BIOSTATISTICAL AND DATA SCIENCE
WAKE FOREST SCHOOL OF MEDICINE
MEDICAL CENTER BOULEVARD
WINSTON-SALEM, NC 27157

XIE, ANPING, PHD
ASSISTANT PROFESSOR
ARMSTRONG INSTITUTE FOR PATIENT SAFETY
DEPT. OF ANESTHESIOLOGY & CRITICAL CARE MEDICINE
JOHNS HOPKINS SCHOOL OF MEDICINE
BALTIMORE, MD 21202

YIN, HSIANG, MD
ASSOCIATE PROFESSOR OF PEDIATRICS
NYU SCHOOL OF MEDICINE
550 FIRST AVENUE, NBV8E14
NEW YORK, NY 10016

ZHENG, KAI, PHD
ASSOCIATE PROFESSOR
DEPARTMENT OF INFORMATICS
DONALD BREN SCHOOL OF INFO. AND COMPUTER
SCIENCES
UNIVERSITY OF CALIFORNIA, IRVINE
IRVINE, CA 92697-3440

ZHU, XI, PHD
ASSOCIATE PROFESSOR
DEPARTMENT OF HELATH MANAGEMENT AND POLICY
THE UNIVERSITY OF IOWA
COLLEGE OF PUBLIC HEALTH
145 N. RIVERSIDE DR. N222 CPHB
IOWA CITY, IA 52242

SCIENTIFIC REVIEW OFFICER

APONTE, BORIS, PHD
DIVISION OF SCIENTIFIC REVIEW
OFFICE OF EXTRAMURAL RESEARCH, EDUCATION
AND PRIORITY POPULATIONS
AGENCY FOR HEALTHCARE RESEARCH AND QUALITY
ROCKVILLE, MD 20857

GAINES, CHRISTOPHER M, MBA
PROGRAM ANALYST
DIVISION OF SCIENTIFIC REVIEW
OFFICE OF EXTRAMURAL RESEARCH, EDUCATION &
PRIORITY POPULATIONS
AGENCY FOR HEALTHCARE RESEARCH & QUALITY
ROCKVILLE, MD 20850

Consultants are required to absent themselves from the room during the review of any application if their presence would constitute or appear to constitute a conflict of interest.
